# High-Resolution Fluorescence Spectra of Airborne Biogenic Secondary Organic Aerosols: Comparisons to Primary Biological Aerosol Particles and Implications for Single-Particle Measurements

**DOI:** 10.1021/acs.est.1c02536

**Published:** 2021-10-26

**Authors:** Minghui Zhang, Hang Su, Guo Li, Uwe Kuhn, Siyang Li, Thomas Klimach, Thorsten Hoffmann, Pingqing Fu, Ulrich Pöschl, Yafang Cheng

**Affiliations:** † Multiphase Chemistry Department, Max Planck Institute for Chemistry, Mainz 55128, Germany; ‡ Minerva Research Group, Max Planck Institute for Chemistry, Mainz 55128, Germany; § Institute for Inorganic and Analytical Chemistry, Johannes Gutenberg University of Mainz, Duesbergweg 10-14, Mainz 55128, Germany; ∥ Institute of Surface-Earth System Science, School of Earth System Science, Tianjin University, Tianjin 300072, China

**Keywords:** airborne bioaerosols, biogenic secondary organic aerosols, real-time detection, autofluorescence, fluorescence spectra, single-particle measurement, aging process

## Abstract

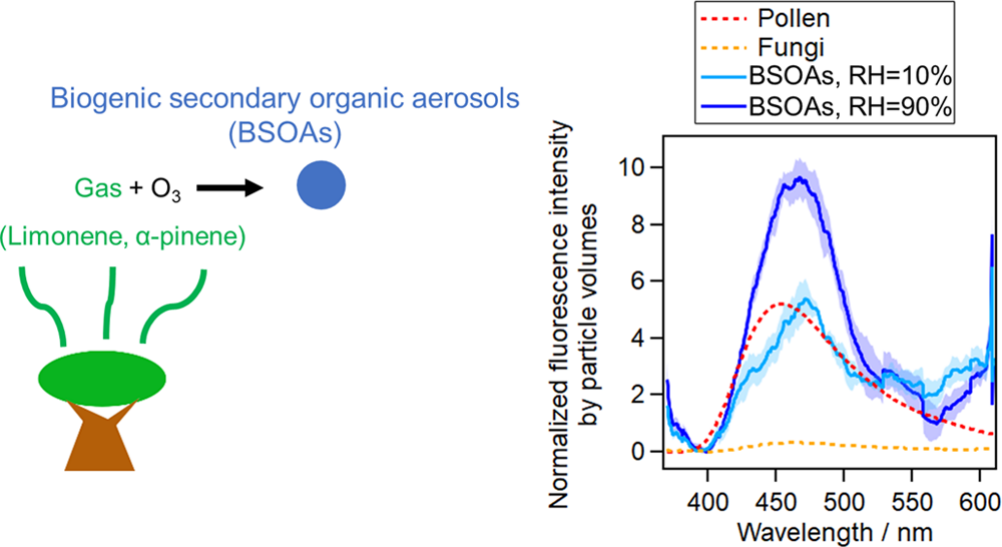

Aqueous extracts of biogenic secondary organic aerosols (BSOAs) have been found to exhibit fluorescence that may interfere with the laser/light-induced fluorescence (LIF) detection of primary biological aerosol particles (PBAPs). In this study, we quantified the interference of BSOAs to PBAPs by directly measuring airborne BSOA particles, rather than aqueous extracts. BSOAs were generated by the reaction of *d*-limonene (LIM) or α-pinene (PIN) and ozone (O_3_) with or without ammonia in a chamber under controlled conditions. With an excitation wavelength of 355 nm, BSOAs exhibited peak emissions at 464–475 nm, while fungal spores exhibited peak emissions at 460–483 nm; the fluorescence intensity of BSOAs with diameters of 0.7 μm was in the same order of magnitude as that of fungal spores with diameters of 3 μm. The number fraction of 0.7 μm BSOAs that exhibited fluorescence above the threshold was in the range of 1.9–15.9%, depending on the species of precursors, relative humidity (RH), and ammonia. Similarly, the number fraction of 3 μm fungal spores that exhibited fluorescence above the threshold was 4.9–36.2%, depending on the species of fungal spores. Normalized fluorescence by particle volumes suggests that BSOAs exhibited fluorescence in the same order of magnitude as pollen and 10–100 times higher than that of fungal spores. A comparison with ambient particles suggests that BSOAs caused significant interference to ambient fine particles (15 of 16 ambient fine particle measurements likely detected BSOAs) and the interference was smaller for ambient coarse particles (4 of 16 ambient coarse particle measurements likely detected BSOAs) when using LIF instruments.

## Abstract 2

An online fluorescence spectrometer for bioaerosol detection suggests distinct contributions of biogenic secondary organic aerosols to fine-mode and coarse-mode fluorescent particles.

## 1 Introduction

Primary biological aerosol particles (PBAPs, also called bioaerosols, e.g., bacteria, viruses,^1,2^ fungi, and pollen) are of great interest to environmental scientists^3−6^ due to their potential influence on climate^7−10^ and human health.^11−15^ Laser/light-induced fluorescence (LIF)^16−23^ techniques have been developed to quantify the concentration of PBAPs in real time and the fractions of PBAPs to total aerosol particles, based on their autofluorescence^24^ after ultraviolet (UV) light excitation. Furthermore, the dispersed fluorescence spectra^25,26^ and the fluorescence lifetime^27^ can potentially be used to classify different types of PBAPs.^28^ In ambient measurements, particles not primarily derived from biological sources can also exhibit fluorescence under UV excitation. For example, it has been demonstrated that combustion-related particles caused interference to the measurements of PBAPs at λ_ex_ = 263 nm^29^ or λ_ex_ = 280 nm.^30^

Biogenic secondary organic aerosols (BSOAs) might exhibit fluorescence and hence interfere with LIF measurements of PBAPs. BSOAs are formed by oxidation of volatile organic compounds such as *d*-limonene (LIM) or α-pinene (PIN) with ozone and radicals.^31−37^ Besides, ammonia might react with *d*-limonene or α-pinene ozonolysis products given that these products are rich in carbonyl groups;^38−40^ as a result, the optical properties such as absorption and fluorescence might change after reacting with ammonia.

It is estimated that BSOAs contribute 90% to total SOAs globally.^41^ A hierarchical cluster analysis of data based on LIF field measurements revealed that a major share of observed fluorescence signals might possibly be attributed to BSOAs.^42^ Indeed, two studies show that BSOAs dissolved in water exhibited sufficient fluorescence to interfere with that of PBAPs.^43,44^

However, the fluorescence characteristics (e.g., emission peaks and intensities) measured for aqueous extracts can differ from those measured for solid particles/powders.^24^ For example, solid riboflavin powders exhibit peak emissions at longer wavelengths than those of aqueous solution.^24^ Another example is that humic acid powders emit very weak fluorescence while humic acid aqueous solution exhibits strong fluorescence.^24^

Moreover, the size of particles needs to be considered during comparisons given that the fluorescence intensity scales with the size of particles.^45,46^ The comparison of BSOAs with PBAPs is not quantitative without the information of particle diameters.

In this study, we directly measured fluorescence properties of airborne BSOA particles by using a recently developed size-resolved single-particle fluorescence spectrometer (S2FS)^47^ (Figure S1) without dissolving them in water. In addition, S2FS enables simultaneous measurements of aerodynamic diameters of particles.^47^ This study aims at quantifying the interference of BSOAs to PBAPs when using LIF instruments. It is essential for correctly characterizing and understanding the online measurements of PBAPs (mostly based on fluorescence signals),^16−23^ which has not been achieved so far in the aerosol and atmospheric community.

In this study, we generated BSOAs in a chamber, measured the fluorescence spectra of BSOAs, and made comparisons with PBAPs. We find that 0.7 μm (diameter) BSOAs caused strong interference to 3 μm (diameter) fungal spores, while the interference was negligible for 10 μm (diameter) pollen. However, normalized fluorescence by particle volumes suggests that BSOAs exhibited fluorescence in the same order of magnitude as that of pollen and 1–2 orders of magnitude higher than fungal spores. Then, we compared the fluorescence spectra of BSOAs with those of ambient particles. It shows that 15 of 16 ambient fine particle measurements likely detected BSOAs, while 4 of 16 ambient coarse particle measurements likely detected BSOAs, which supports the common practice that the data of fine particles were deleted when using LIF instruments.^16−23^ In the end, we discussed implications for single-particle fluorescence measurements, especially for real-world monitoring.

## 2 Materials and Methods

### 2.1 Generation of BSOAs

The reaction of ozone (O_3_) with monoterpene is one of the commonly used standard approaches to generate BSOAs.^48−50^ Limonene and α-pinene have been extensively used to study the formation of BSOAs^48−50^ as limonene accounts for 16% and α-pinene accounts for 50% of monoterpene emissions globally.^41^ So we also used limonene and α-pinene as representative precursors for BSOA formation. In this study, the formation of BSOAs was carried out in dark conditions to avoid the complex effects of radiation given that radiation has two contradictory impacts on the fluorescence of BSOAs. On the one hand, radiation induced photochemical reactions to possibly increase the fluorescence;^51,52^ on the other hand, radiation bleached molecules to decrease the fluorescence.^53,54^

BSOAs were generated in a ∼0.7 m^3^ humidified smog chamber made of Teflon film (50 μm wall thickness), which was similar to the method by Lee et al.^44^ O_3_ was flushed through the chamber overnight to allow for wall surface passivation, which resulted in a final concentration of ∼400 ppb in the reaction chamber. O_3_ was generated by photolysis of O_2_ using an ozone generator based on UV light (Type SOG-2, Analytik Jena AG, Jena, Germany). O_3_ concentrations were measured using an O_3_ analyzer (Model 49*i*, Thermo Fisher Scientific, USA). Then the outlets of the chamber were closed and a small internal fan was activated to achieve turbulent mixing. Then, 2 μL of *d*-limonene or α-pinene was flushed into the chamber by means of compressed aerosol-free air with a flow rate of 4 LPM for 2 min, corresponding to a concentration of ∼400 ppb if not accounting for any chemical or wall loss. After 3 min of mixing using a fan, static conditions in the dark prevailed for another ∼20 h to allow for BSOA formation.

Ammonia can react with the BSOAs, which is called ammonia-mediated aging process. In our study, the approach to mimic an ammonia-mediated chemical aging effect on BSOAs is somewhat different from earlier reports.^44^ Instead of collecting BSOAs on filters, followed by exposure to ammonia, we supplied the smog chamber with ammonia in addition to O_3_, prior to flushing *d*-limonene or α-pinene into the chamber. In detail, after flushing with O_3_, the compressed air passed the surface of ammonia solution (0.18 M) with an air flow rate of 0.5 LPM for 5 min. The concentration of ammonia in the chamber was ∼50 ppm according to the measurement by cavity-ring-down spectroscopy (Picarro, Santa Clara, CA, USA). Then, 2 μL of *d*-limonene or α-pinene was flushed into the chamber with compressed air with a flow rate of 4 LPM for 2 min. After 3 min of mixing using a fan, the reaction process prevailed statically for ∼20 h in the dark.

According to Atkinson et al.,^55^ the gas phase reactions of O_3_ of with *d*-limonene or α-pinene produced OH radicals with yields of 0.86 and 0.85, respectively. Therefore, the SOA formed was a mixture of O_3_ and OH radical reaction products. The rate coefficient of OH/NH_3_ was 1.47 × 10^–13^ cm^3^ mol^–1^ s^–1^ and the rate coefficient of OH/NH_2_ was 7 × 10^–12^ cm^3^ mol^–1^ s^–1^ at room temperatures,^56^ which were much lower than the rate coefficient of OH/α-pinene of 5.1 × 10^–11^ cm^3^ mol^–1^ s^–1^ and the rate coefficient of OH/limonene of 1.6 × 10^–10^ at room temperature.^57^ However, the concentration of ammonia was two orders of magnitude higher than that of monoterpene in our chamber experiment. Therefore, we cannot rule out direct involvement of ammonia with hydroxyl radical and related products, namely, fluorescence properties of BSOAs could be affected by these nitrogen-containing compounds.

The observed number size distribution of BSOAs showed a peak at ∼200 nm, but the lower detection limit of the S2FS for particles was ∼500 nm. Particles with diameters of 200 nm cannot be measured by the S2FS, but these small particles can contaminate the nozzle surface inside the optics chamber of the S2FS given their much higher number concentration compared with the large particles. Therefore, we applied a differential mobility analyzer (DMA) at the smog chamber outlet to select only large particles. The sheath flow of the DMA was relatively low at 2 LPM, and the aerosol flow was 1 LPM. We intentionally used the flow ratio of 2 to obtain high number concentration of BSOAs. For a DMA-CPC (condensation particle counter) system, such an aerosol-sheath flow ratio is not optimal for sizing. However, our system can directly measure the aerodynamic diameter of aerosols as an aerodynamic particle sizer (APS), and thus do not strongly rely on the sizing of DMA. Otherwise, the background noise of the detector is too large when the number concentration of particles is low.

With this size setting and the flow rate setting, both the number concentrations (Figure S2) and fluorescence signals of particles were found to be high enough for particle fluorescence measurements. The S2FS was directly connected to the outlet of the DMA (Figure S1). The particle diameter was set to be 800 nm in DMA, and the aerodynamic diameter was measured with the peak at ∼700 nm by the S2FS (Figure S2).

### 2.2 PBAP Measurements and Ambient Measurements

Malt petri dishes (VWR International) were used to culture fungal spores for 4 weeks. The fungal spores were flushed in a glass chamber with compressed air; the S2FS was connected to the other outlet of the glass chamber.

The ambient measurements were conducted from May 31 to June 8, 2017, on the roof of the Max Planck Institute for Chemistry in the daytime, which is located at the semirural area in Central Europe. The details of fungal spore measurements and ambient measurements can be found in our previous paper.^47^ The data were reanalyzed here to do comparisons to BSOAs.

### 2.3 Construction of the S2FS

The technical setup and performance of the instrument were described in detail in our previous paper.^47^ In brief, the aerosol flow was 1 LPM. The excitation wavelength (λ_ex_) is 355 nm and the measured fluorescence emission is from 370 to 610 nm dispersed in 512 channels, where a major portion of the fluorescence of PBAPs happens.^24^ The S2FS can measure aerodynamic diameters when particles flow through two red-laser beams and directly measure fluorescence properties of aerosol particles when particles flow through the UV-laser beam. On the single-particle level, the fluorescence spectra are nearly complete for highly fluorescent particles such as pollen.^47^ But for weakly fluorescent particles such as fungal spores, signals only appear randomly on a few pixels for the single particle and averaging over 100 to 5000 particles is needed to get complete spectra.^47^

### 2.4 Definition of Fluorescence Index and Fluorescence Sharpness

The fluorescence index (FI, which was the ratio of fluorescence intensity at 450 nm to that at 500 nm) was a common method to characterize different types of organic matter. For example, the FI of microbially derived fulvic acids was smaller than that of terrestrially derived fulvic acids, which implies that the source of organic matter in the water can be distinguished based on the FI.^58,59^

According to our observations, the peak position of BSOA particles was located at ∼470 nm, rather than at ∼450 nm. Therefore, we defined a new term fluorescence sharpness (FS), which was calculated as the ratio of the fluorescence intensity at 470 nm to that at 500 nm.

## 3 Results and Discussion

### 3.1 Fluorescence Spectra and Intensity of BSOAs

**Figure 1:**
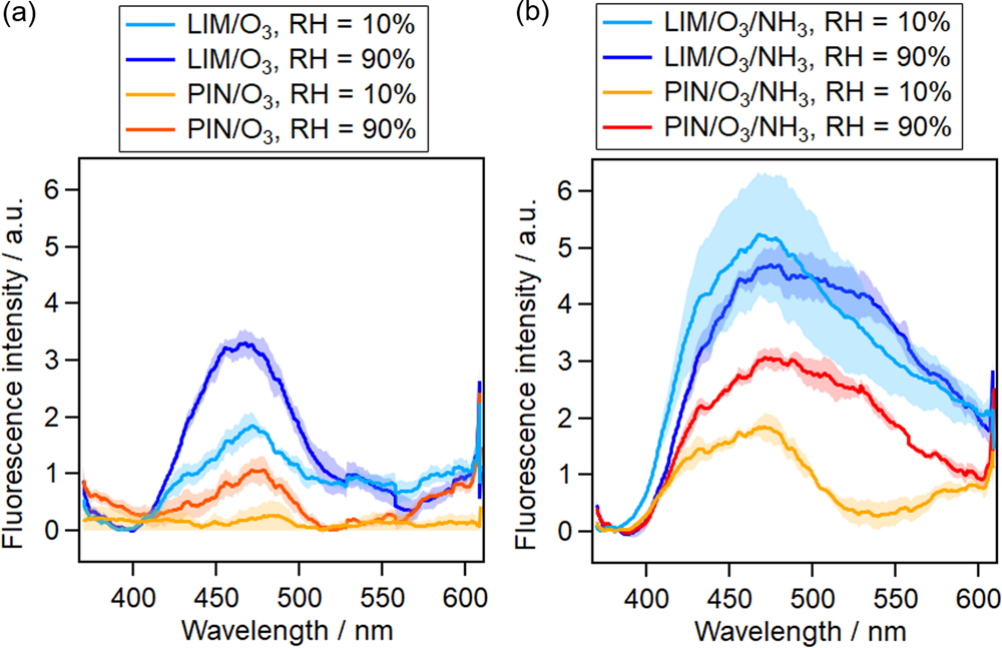
Averaged fluorescence spectra of BSOA particles (a) without ammonia and (b) with ammonia during BSOA formation, which were based on over four groups of particles and smoothed over 64 pixels. Each group consisted of 5000 particles. The shade areas indicate standard deviations based on four groups. The RH and ammonia affect the intensity and shape of the fluorescence spectra in a cooperative way.

On a single-particle level, fluorescence signals only appeared on a few pixels (Figure S3). In order to obtain complete and reproducible spectra, we averaged fluorescence signals of 5000 BSOA particles. Figure 1a shows that LIM/O_3_-generated SOAs (relative humidity (RH) = 10%) and PIN/O_3_-generated SOAs (RH = 10%) exhibited peak emissions at nearly the same wavelength of ∼470 nm with the excitation wavelength of λ_ex_ = 355 nm; the fluorescence intensity of LIM/O_3_-generated SOAs (RH = 10%) was ∼4 times higher than that of PIN/O_3_-generated SOAs (RH = 10%). At a higher RH, the peak shifted to a shorted wavelength of 464 nm for LIM/O_3_-generated SOAs (RH = 90%) while it shifted to a longer wavelength of 474 nm for PIN/O_3_-generated SOAs (RH = 90%). The fluorescence intensity increased by a factor of 2–5 at higher RH.

If ammonia was added during BSOA formation, the fluorescence intensity and spectral shape were affected significantly (Figure 1b). In the absence of ammonia, a general increase in fluorescence intensity was observed at a higher RH to happen predominantly in the wavelength range of the peak emissions (Figure S4a,b). Meanwhile, in the presence of ammonia, the increased RH also has considerable effects on the shape of the fluorescence spectra in the longer wavelength range (Figure S4c,d). Similarly, the comparison of the fluorescence spectra of BSOAs indicates that water was also important in leading to increased fluorescence response at longer wavelengths (for direct comparison, see the rearranged data set in Figure S5). Enhanced fluorescence emissions at a longer wavelength were obvious if both conditions—a high RH in the presence of ammonia—apply and therefore can be explained by cooperative effects of water content and ammonia. The concentration of ammonia we used here was ∼50 ppm, which was much higher than 1–54 ppb in ambient measurement conditions.^39,40^ Therefore, the above effect of ammonia can be regarded as the upper limit.

Apart from the peak positions, the fluorescence index (FI, which is the ratio of fluorescence intensity at 450 nm to that at 500 nm) was an alternative method to characterize different types of organic matter. In our case, freshly formed SOA particles had a higher FI than the corresponding ammonia-mediated aged SOA particles (Table 1). One exception was that PIN/O_3_-generated SOAs (RH = 10%) had a lower FI than PIN/O_3_/NH_3_-generated SOAs (RH = 10%). The reason is that BSOAs exhibited peak emissions at ∼470 nm, rather than at ∼450 nm.

**Table 1:** Fluorescence Peak Positions, Fluorescence Index (FI), and Fluorescence Sharpness (FS) for SOA and Fungal Spores

	peak positions	FI (450 nm/500 nm)	FS (470 nm/500 nm)
LIM/O_3_, RH = 10%	471 nm (±3 nm)	1.22 ±0.32	1.74 ±0.44
LIM/O_3_, RH = 90%	464 nm (±6 nm)	1.63 (±0.16)	1.82 (±0.21)
PIN/O_3_, RH = 10%	470 nm (±12 nm)	1.38 (±0.34)	3.21 (±2.12)
PIN/O_3_, RH = 90%	474 nm (±5nm)	1.64 (±0.49)	2.88 (±0.97)
LIM/O_3_/NH_3_, RH = 10%	471 nm (±5nm)	1.06 (±0.12)	1.18 (±0.11)
LIM/O_3_/NH_3_, RH = 90%	475 nm (±6 nm)	0.9 (±0.08)	1.05 (±0.11)
PIN/O_3_/NH_3_, RH = 10%	470 nm (±4 nm)	1.56 (±0.12)	1.83 (±0.19)
PIN/O_3_/NH_3_, RH = 90%	473 nm (±5 nm)	0.91 (±0.07)	1.11 (±0.13)
*Cladosporium cladosporioides*	460 nm	1.29	1.37
*Aspergillus versicolor*	468 nm	1.18	1.37
*Cladosporium herbarum*	483 nm	0.69	0.98
*Penicillium chrysogenum*	474 nm	1.32	1.55

As the peak position of BSOAs was located at ∼470 nm, we defined the fluorescence sharpness (FS) as the ratio of the fluorescence intensity at 470 nm to that at 500 nm. All of the freshly formed SOA particles exhibited a much higher FS than that of the ammonia-mediated aged particles (Table 1). Therefore, the FS might be a better parameter than the FI in characterizing BSOA particles. Note that FS has not been applied broadly yet to characterize aerosol particles and thus needs further investigations in the future.

The pattern of BSOA particles at λ_ex_/λ_em_ = 355 nm/470 nm was similar to the fluorescence of humic-like substance (HULIS) at λ_ex_/λ_em_ = 355 nm/464 nm as observed in ambient measurements,^35^ suggesting that BSOAs contain compounds with similar fluorescence properties to those of HULIS. Note that airborne BSOA particles were directly measured here, while HULIS was normally measured in the aqueous phase.^35^

Compared with the aqueous-phase BSOA extracts measured by Lee et al.,^44^ airborne BSOA particles measured revealed peak fluorescence emissions at longer wavelengths of 20 to 34 nm. One possible reason is that in the liquid phase, fluorescence is directly emitted, while in the solid phase, the light emitted may be reabsorbed and re-emitted, resulting in a shift of light emission to longer wavelengths.^18^ The other possible explanation is that ammonia-mediated aging of BSOA particles was conducted with a higher ammonia concentration of ∼50 ppm here, which was much higher than that of ∼100 ppb used by Lee et al.^44^ Hence the final chemical composition might also be different.

Note that the above results only include LIM/O_3_-generated SOAs and PIN/O_3_-generated SOAs, which do not necessarily represent all of SOAs. Other species of BSOAs and anthropogenic SOAs were not tested in this study. In addition, these BSOAs were generated under experimental controlled conditions. The above findings observed in controlled conditions in chambers do not necessarily represent real-world observations.

### 3.2 Possible Mechanisms for BSOA Fluorescence

PIN/O_3_-generated and LIM/O_3_-generated SOAs are complex mixtures of numerous, functionalized organic compounds.^48−50^ Both systems contain nonconjugated oxygenated compounds that enable light absorption at 355 nm.^60^ The imaginary part of the complex refractive index of PIN/O_3_-generated SOAs was very low at 355 nm,^61−63^ which explained weak absorption at 355 nm^62^ and hence weak fluorescence. Unexpectedly, LIM/O_3_-generated SOAs exhibited stronger fluorescence even though the imaginary part of LIM/O_3_-generated SOAs was smaller than that of PIN/O_3_-generated SOAs at 355 nm.^63^ This suggests that LIM/O_3_-generated SOAs had larger quantum yield (ratio of the number of photons emitted to the number of photons absorbed) than that of PIN/O_3_-generated SOAs.

If NH_3_ was added during BSOA formation, it is reasonable to assume that ammonia reacted with organic acids in the BSOAs to form salts.^50^ The increased fluorescence of NH_3_-mediated aged BSOAs could be attributed to polycarbonyl/NH_3_ reactions that form highly conjugated nitrogen-containing aromatic heterocyclic imine compounds.^43,64−66^

RH affects BSOA formation and aging processes in several ways. First, RH affects the phase state and thus the diffusion processes in BSOAs and gas-particle partitioning, changing the final chemical composition.^67^ Second, RH affects aerosol water content and hence solute concentrations and aqueous-phase reaction rates.^68,69^ Third, RH in the presence of NH_3_ affects the acidity (pH) of aerosols, changing their physical and chemical behavior,^70,71^ which is related to the BSOA formation and optical properties. Although the nitrogen-containing chromophores contributed only ∼2% to total aerosol mass of the aerosol in the presence of NH_3_,^72^ these nitrogen-containing chromophores significantly affected the shape of the fluorescence. The shift of fluorescence to longer wavelengths due to cooperative effects of RH and NH_3_ suggests a higher degree of aromaticity or a more functionalized composition.^43,73,74^

### 3.3 Comparisons with PBAPs

**Figure 2:**
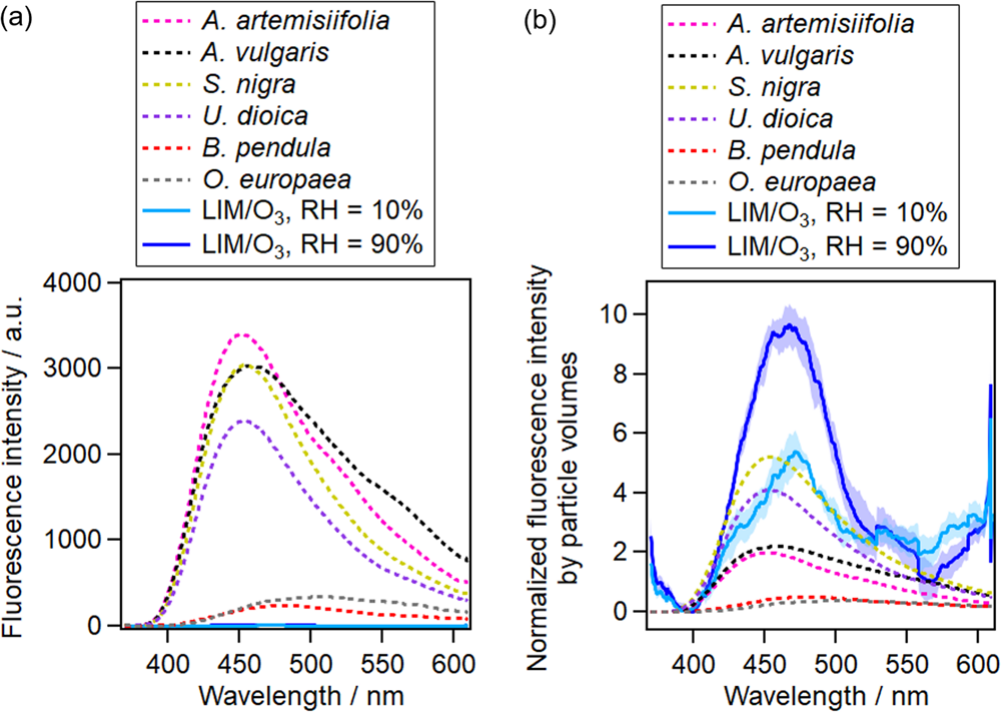
Averaged fluorescence spectra of six species of pollen (diameter 7–18 μm) versus 0.7 μm (diameter)LIM/O_3_-generated SOAs (a) before normalization and (b) after normalization of fluorescence by particle volumes. The spectra were smoothed over 64 pixels. BSOAs consisted of 5000 particles and pollen consisted of 300 particles for averaging.

The fluorescence intensity of pollen was 100–1000 times higher than that of BSOAs,^47^ which makes the interference of BSOAs to pollen negligible (Figure 2a). However, after normalizing the fluorescence intensity by particle volumes, four species of pollen exhibited fluorescence in the same order of magnitude as that of LIM/O_3_-generated SOAs (RH = 10%) (Figure 2b). The other two species of pollen (*B. pendula*, *O. europaea*) exhibited fluorescence which was 10 times lower than that of LIM/O_3_-generated SOAs (RH = 10%) (Figure 2b). These results suggest that the reason for the weak fluorescence of BSOAs compared to pollen is their particle size (number of fluorescent molecules), rather than the absorption property or quantum yield.

**Figure 3:**
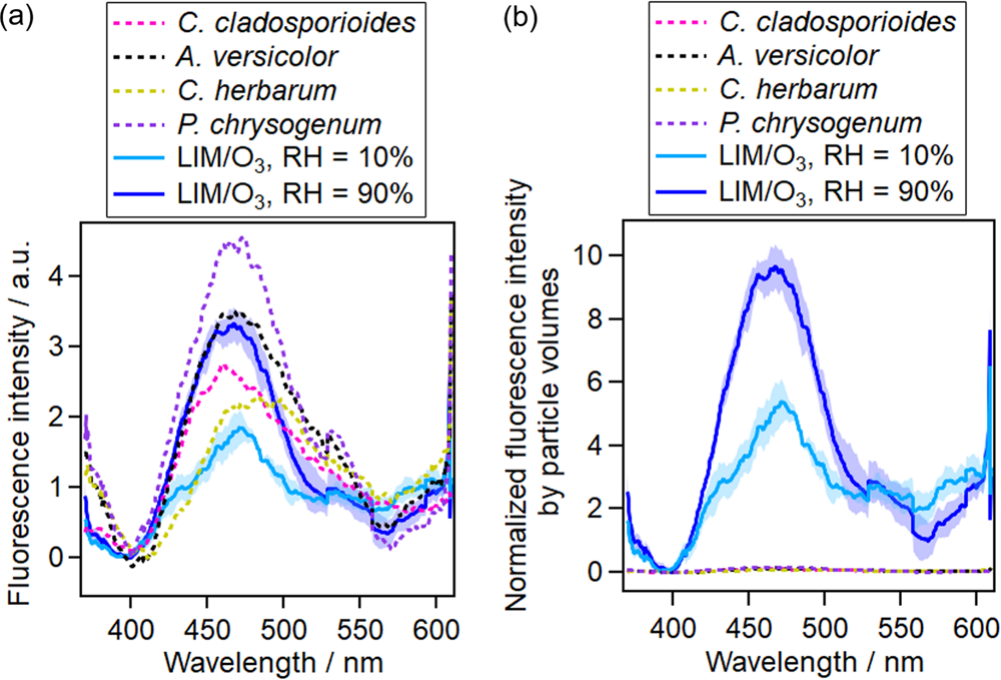
Averaged fluorescence spectra of four species of fungal spores (diameter 2–4 μm) versus 0.7-μm (diameter) LIM/O_3_-generated SOAs (a) before normalization and (b) after normalization of fluorescence by particle volumes. The spectra were smoothed over 64 pixels. BSOAs consisted of 5000 particles and fungal spores consisted of 3000 particles for averaging. Both the fluorescence intensity and fluorescence shape exhibited similar characteristics for fungal spores and BSOAs.

The fluorescence intensity of fungal spores (diameters from 2 to 4 μm) was in the same order of magnitude as that of 0.7 μm BSOAs (Figure 3a). Also the fungal spore peak emission wavelengths of 460–483 nm resembled those of BSOAs at 464–475 nm (Table 1).

In addition, the FS of fungal spores was in the range of 0.98–1.55, which fell into the similar range of 1.05–3.21 for BSOAs. Therefore, BSOA particles or particles coated with biogenic BSOAs might interfere with measurements of PBAPs such as fungal spores (Table 1).

Unexpectedly, after normalizing the fluorescence by particle volumes, the fluorescence of fungal spores was ∼100 times lower than that of LIM/O_3_-generated SOAs (Figure 3b). The imaginary part of LIM/O_3_-generated SOAs is ∼10^–4^, and the imaginary part of PBAPs is also ∼10^–4^,^63,75^ suggesting that absorption is not responsible for the large difference in fluorescence. Therefore, we suppose BSOAs has a much higher quantum yield than that of fungal spores.

Although we did not measure bacteria with the S2FS, the fluorescence intensity of bacteria was in the same order of magnitude as that of fungal spores according to Savage et al.^76^ So our results about fungal spores might also apply to the results for bacteria.

### 3.4 Comparisons with Ambient Fine and Coarse Particles

**Figure 4:**
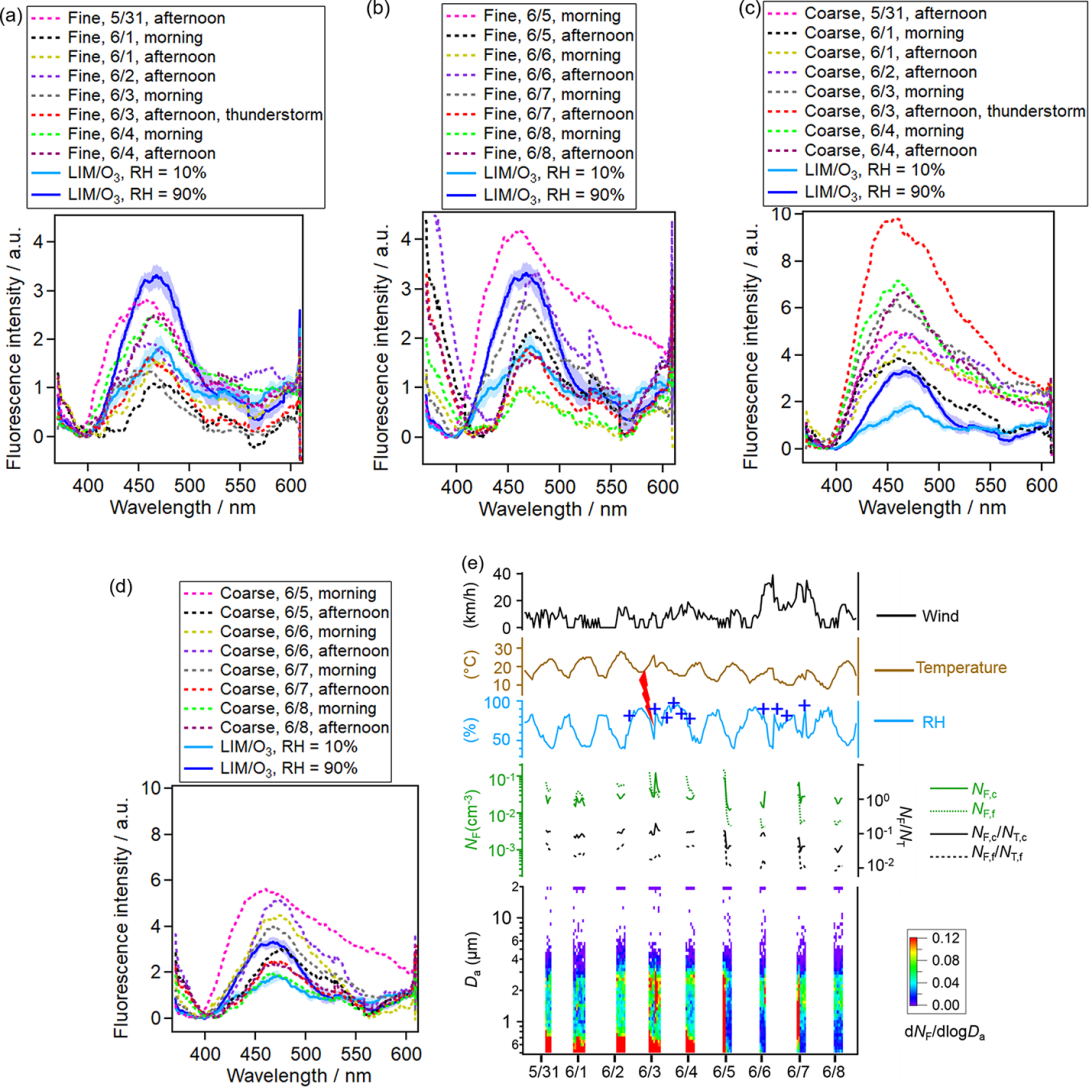
Averaged fluorescence spectra of (a) ambient fine particles (diameter from 0.5 to 1 μm) from 31 May to 4 June, (b) ambient fine particles from 5 June to 8 June, (c) ambient coarse particles (diameter from 1 to 4 μm) from 31 May to 4 June, and (d) ambient coarse particles from 5 June to 8 June. Note that the scale on the *y*-axis of fluorescence intensity was different for fine and coarse particles here. (e) The meteorological conditions and the fluorescent aerosol particle concentrations. The integrated fluorescent aerosol particle number (*N*_F_) and the number ratio of integrated fluorescent (*N*_F_) to total aerosol particles (*N*_T_) are shown in the middle, and the fluorescent aerosol particle number size distribution (d*N*_F_/dlog*D*_a_) is shown in the bottom. The blue crosses indicated rain events, and the red scar indicated the thunderstorm. The major tick marks on the *x*-axis of panel (e) represented 12:00 at noon. In panels (a) to (d) here, all of the particles were averaged; while in the previous paper, only the particles with fluorescence signals above the background threshold were averaged.^47^ Panel (e) shown here is reproduced from the previous paper.^47^

Previous field measurements in Central Europe by Huffman et al.^16^ and our group^47^ suggested that BSOAs might interfere the measurement of fine particles (diameter below 1 μm). Figure 4a,b shows that most of the fluorescence spectra of fine particles (diameter 0.5–1 μm) overlap (15 of 16 fine particle measurements) with the spectra of BSOAs. One exception was the fluorescence spectrum in the morning of 5 June, which exhibited higher fluorescence intensity and broad emissions at longer wavelengths. The fine particles in the morning of 5 June might be PBAPs (bacteria or fungi fragments) as the strong fluorescence only appeared in the morning of 5 June and disappeared in the afternoon of 5 June (Figure 4b).

Figure 4c,d shows that the interference of BSOAs to coarse particles (diameter from 1 to 4 μm) was smaller (4 of 16 coarse particle measurements overlap). Among the four fluorescence spectra of ambient coarse particles that overlap, three appeared in the afternoon and only one appeared in the morning, suggesting that the interference for coarse particles decreased in the morning. The extremely strong fluorescence happened during the thunderstorm in the afternoon of 3 June (Figure 4e). These coarse particles were probably subpollen particles (SPPs), as pollen ruptured into fragments during the thunderstorm, which exhibited a high number concentration of ∼0.1 cm^–3^. Note that this exceptional observation in the afternoon of 3 June only appeared once during the measurements and may need further investigation.

### 3.5 Implications for Single-Particle Measurements

Most of the LIF instruments analyze the integrated fluorescence intensity,^16−22^ rather than the dispersed fluorescence spectrum. Here we used the same background threshold setting as other groups,^16,77^ namely the average plus 3σ. If the fluorescence intensity (i.e., the number of photons) exceeded this threshold, the particle was considered as a fluorescent particle. Highly fluorescent PBAPs such as pollen emitted ∼1000 photons for one single particle, and the signal of every single pollen grain was above the background threshold.^47^ The fluorescence counting efficiency of the S2FS can reach ∼100% for highly fluorescent 10 μm (diameter) pollen.^47^ However, although the S2FS has been demonstrated to be more sensitive than other commercial LIF instruments, for relatively weakly fluorescent particles such as 3 μm (diameter) cellulose particles, only ∼50% of particles were above the background threshold.^47^

**Figure 5:**
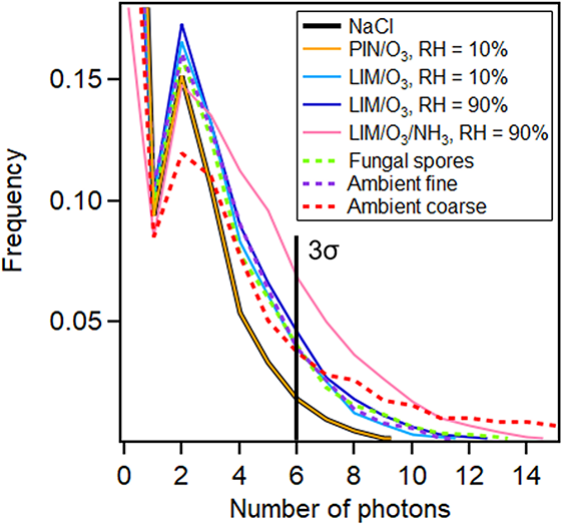
Distribution of the number of photons (fluorescence intensity) for single BSOA particles, fungal spores, and ambient fine (in the afternoon of 31 May) and ambient coarse (in the afternoon of 3 June) particles. The vertical black line indicates the background threshold, which is the average plus 3σ as measured by NaCl. Even for NaCl, 1.9% of particles were above the background threshold due to the stray light of the instrument itself.

For weakly fluorescent PBAPs such as fungal spores, only 36.2% of *P. chrysogenum* fungal spores were above the background threshold (Figure 5). This number decreased to 18.6%, 7.2%,and 4.9% for *A. versicolor*, *C. cladosporioides*, and *C. herbarum*, respectively. The S2FS cannot detect fungal spores with 100% efficacy because (1) a single fungal spore emitted only a few photons as compared to 1000 photons for one single pollen grain; (2) some photons were lost in the optical fiber, focus mirror, and grating mirror; and (3) the detector transferred the photon signals to electronic signals with broad distributions when the number of photons was only a few.

Real-world ambient fine particles (in the afternoon of 31 May) had a fraction of 6.1% and ambient coarse particles (in the afternoon of 3 June) had a fraction of 20.7% above the background threshold. The fraction of fluorescent ambient particles might vary depending on the location and time.

PIN/O_3_-generated SOAs at RH = 10% had a fraction of 1.9% above the background threshold, which was the same as that of NaCl (Figure 5). This fraction increased to 5.5% for LIM/O_3_-generated (RH = 10%) SOAs. At a higher RH = 90%, the fraction increased to 7.2%. In the presence of ammonia, the fraction further increased to 15.9%. These results were consistent with the above results of the averaged fluorescence spectra of particles; that is, for particles with higher averaged fluorescence intensities, the number fraction of particles above the background threshold was also larger.

Using LIF for measurements of PBAPs, fine particles would be more strongly affected (15 of 16 measurements could be BSOAs) than the coarse particles (4 of 16 measurements could be BSOAs). Thus, LIF-based PBAP measurements should be treated with great care in certain environments, especially for fine particles. Pollen can be distinguished from BSOAs with high confidence. Normalized fluorescence by particle volumes suggests that pollen exhibited fluorescence in the same order of magnitude as that of BSOAs, suggesting that the strong fluorescence of pollen is due to its large size. No significant difference of fluorescence spectra was observed between BSOAs and fungal spores in terms of fluorescence peak positions, FI, and FS. Unexpectedly, normalized fluorescence by particle volumes also suggests that the fluorescence intensity of BSOAs was 10–100 times higher than that of fungal spores. This means pure BSOAs exhibited much stronger fluorescence than pure fungal spores of the same size. If BSOAs were coated on non-fluorescent particles such as dust, then distinguishing them from fungal spores is impossible based on the fluorescence intensity.

Apart from fluorescence spectra, fluorescence lifetime,^17,27,78^ multiphoton excitation,^79,80^ dyeing with external fluorophores,^81,82^ and morphology of aerosol^83,84^ might help better distinguish BSOAs from PBAPs and even distinguish between different types of PBAPs, which needs to be further explored in future studies.

## Supporting Information Available

Additional information about technical details and particular topics (PDF)

## Abbreviations

a.u.: arbitrary units
BSOAs: biogenic secondary organic aerosols
FI: fluorescence index
FS: fluorescence sharpness
LIF: laser/light-induced fluorescence
LIM: *d*-limonene
PBAPs: primary biological aerosol particles
PIN: α-pinene
RH: relative humidity
S2FS: size-resolved single-particle fluorescence spectrometer
SOAs: secondary organic aerosols
SPPs: subpollen particles
λ_ex_: excitation wavelength
λ_em_: emission wavelength
