# Supplementary material for: High-Resolution Fluorescence Spectra of Airborne Biogenic Secondary Organic Aerosols: Comparisons to Primary Biological Aerosol Particles and Implications for Single-Particle Measurements
Source: Environ Sci Technol. 2021 Oct 26;55(24):16747–56. doi: 10.1021/acs.est.1c02536 (PMC8697557; doi:10.1021/acs.est.1c02536)
Supplement: Supplementary file 1 — es1c02536_si_001.pdf [file es1c02536_si_001.pdf]

Supporting Information for

High-resolution Fluorescence Spectra of Airborne Biogenic Secondary Organic Aerosols: Comparisons to Primary Biological Aerosol Particles and Implications for Single-particle Measurements

Minghui Zhang,[†,Δ] Hang Su,[*,†] Guo Li,[†] Uwe Kuhn,[†] Siyang Li,[†] Thomas Klimach,[†] Thorsten Hoffmann,[¶] Pingqing Fu,[§] Ulrich Pöschl,[†] and Yafang Cheng[‡]

[†]Multiphase Chemistry Department, Max Planck Institute for Chemistry, 55128 Mainz, Germany

[‡]Minerva Research Group, Max Planck Institute for Chemistry, 55128 Mainz, Germany

[¶]Institute for Inorganic and Analytical Chemistry, Johannes Gutenberg University of Mainz, Duesbergweg 10-14, Mainz 55128, Germany

[§]Institute of Surface-Earth System Science, School of Earth System Science, Tianjin University, Tianjin 300072, China

[Δ] Present address: Université Clermont Auvergne, CNRS, SIGMA Clermont, Institut de Chimie de Clermont-Ferrand, Clermont-Ferrand 63000, France

Corresponding Author

[*]H. Su, Phone: +49 (0) 61313057300; e-mail: h.su@mpic.de

Supporting Information includes 8 pages and 6 figures.

Experimental setup

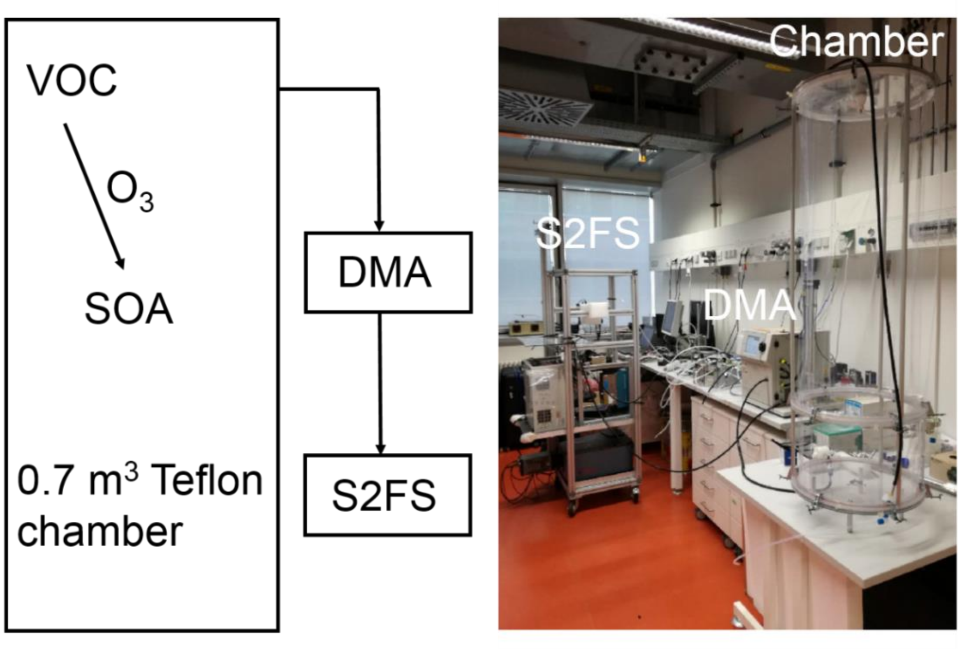

Figure S1. Construction of the smog chamber and the size-resolved single-particle fluorescence spectrometer (S2FS). The SOA particles were size-selected by a differential mobility analyzer (DMA), and the fluorescence properties of the aerosol were directly measured by the S2FS.

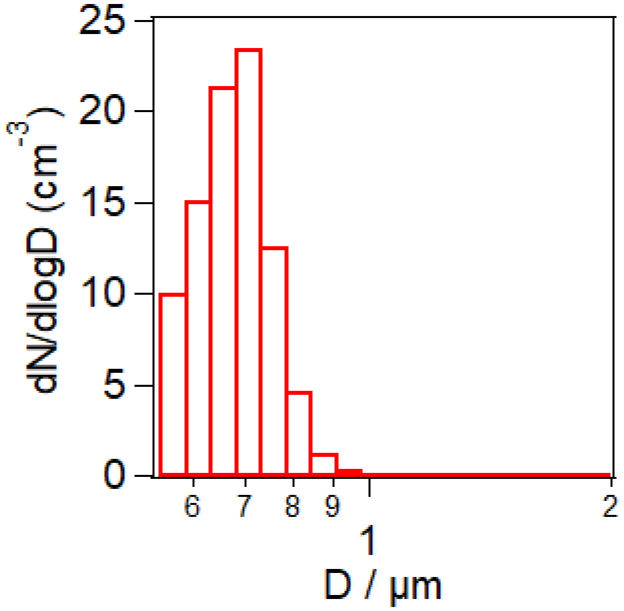

Figure S2. The number size distribution of SOA particles (LIM/O_3 at RH = 90%). The peak of the aerodynamic diameter was ~0.7 μm when the diameter of DMA was set as 0.8 μm.

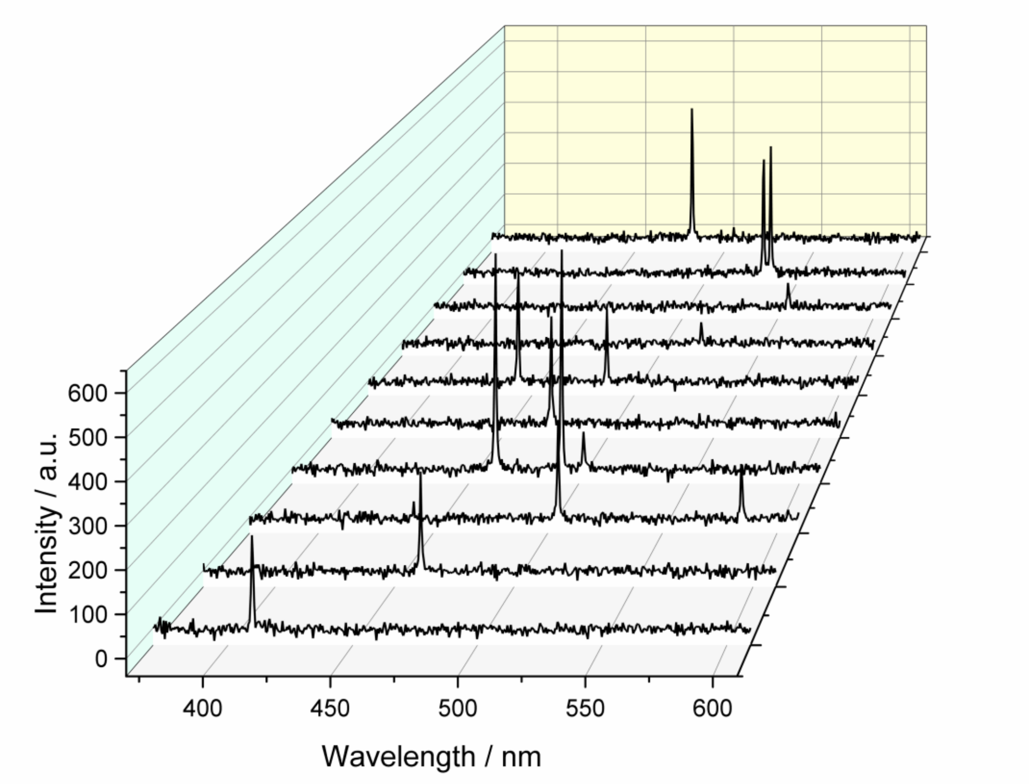

Figure S3. Individual spectra of the single particle of SOA (LIM/O_3 at RH = 90%). The signals of one individual SOA particle appeared only on a few pixels as one single SOA only emitted a few photons. In comparison, one single pollen grain can emit ~1000 photons and the spectrum is nearly complete for one single pollen grain.[1]

Cooperative effect of ammonia and the relative humidity (RH) on the fluorescence spectra

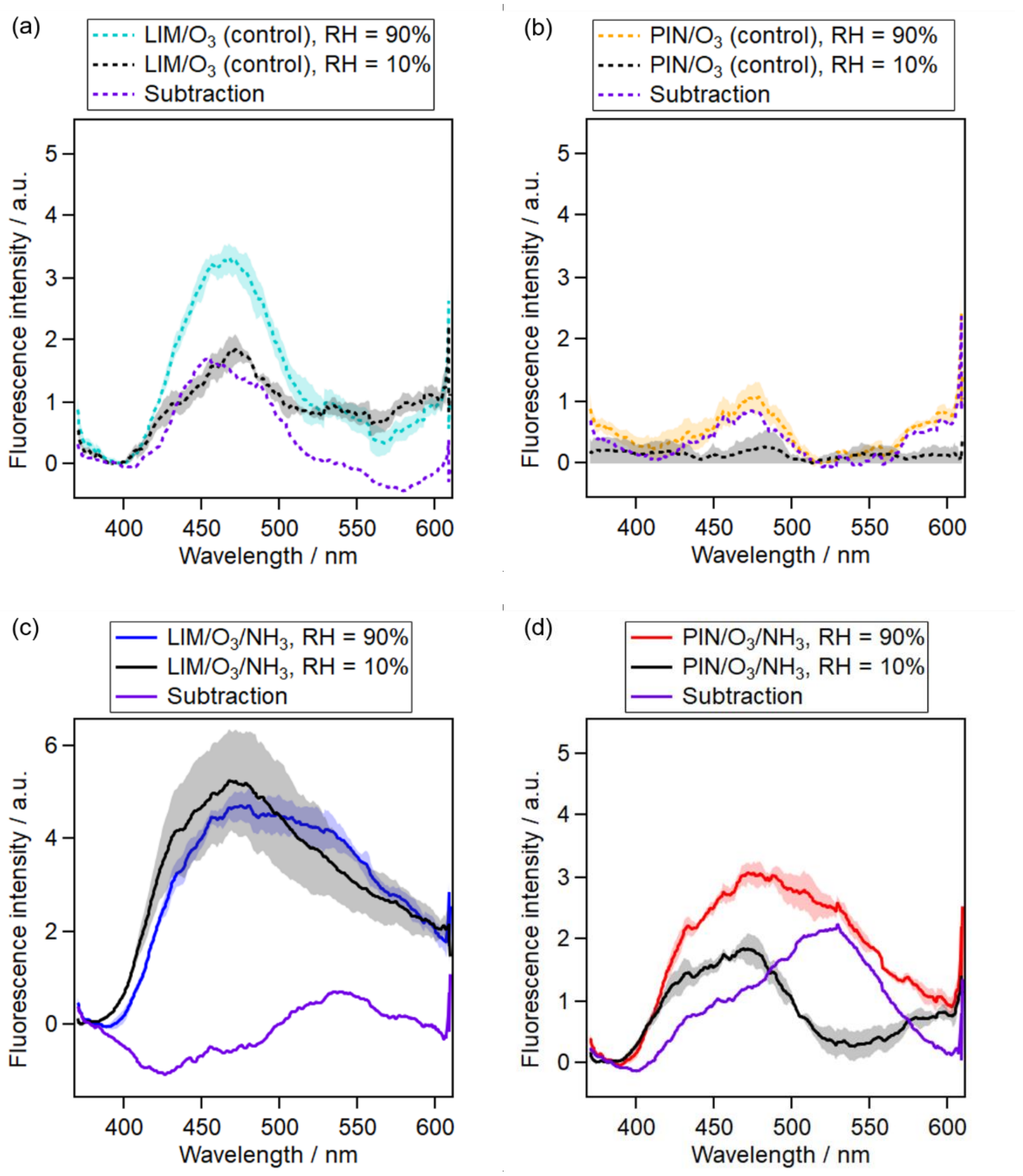

Figure S4. Fluorescence spectra of SOA particles at different RH, which were averaged over 4 groups of particles and smoothed over 64 pixels. Each group consists of 5000 particles. The shades areas indicate standard deviations. Purple lines (subtraction) show the fluorescence at RH = 90% minus that at RH = 10%, which suggest cooperative effects of ammonia and water on the fluorescence at longer wavelengths.

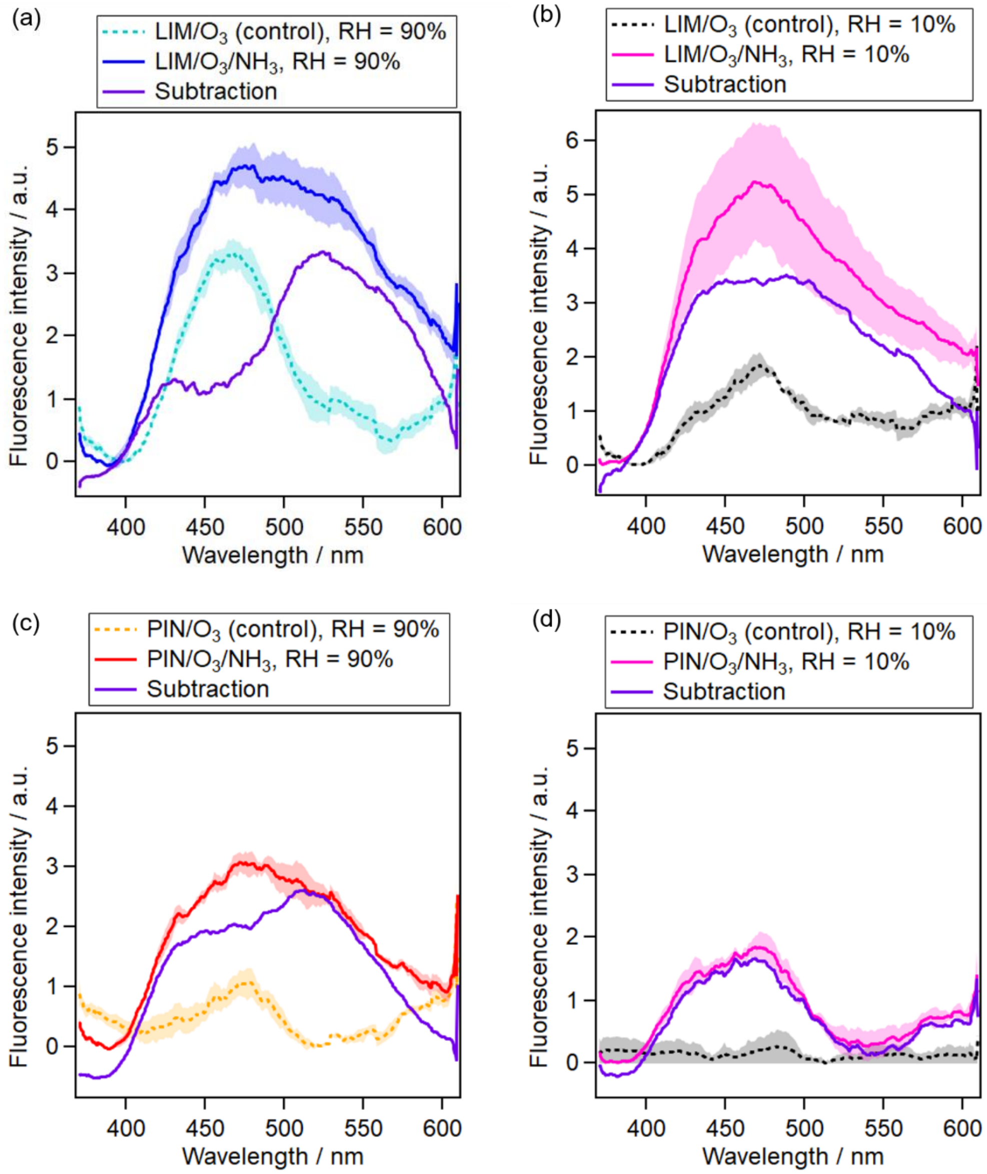

Figure S5. Fluorescence spectra of SOA particles, which were averaged over 4 groups of particles and smoothed over 64 pixels. Each group consists of 5000 particles. The shades areas indicate standard deviations. Purple lines (subtraction) show the fluorescence in the presence of ammonia minus that in the absence of ammonia (control), which suggest cooperative effects of ammonia and water on the fluorescence at longer wavelengths.

Effect of the Nafion dryer on the fluorescence spectra

The other reason for the change in fluorescence with different RH could be that the RH influences the physical hydration state of particles. However, during our measurements, the sheath flow of DMA and the S2FS was from lab room air. This way, the RH inside the optics chamber of the S2FS was 32% to 48% during the overall measurements, regardless of the RH of the reaction smog chamber. Even if a Nafion dryer was added in front of the S2FS, no obvious change was observed for the fluorescence spectra of LIM/O_3-generated SOA particles (Figure S6). Therefore, the change of fluorescence spectra at different RH is not due to the physical hydration state of particles.

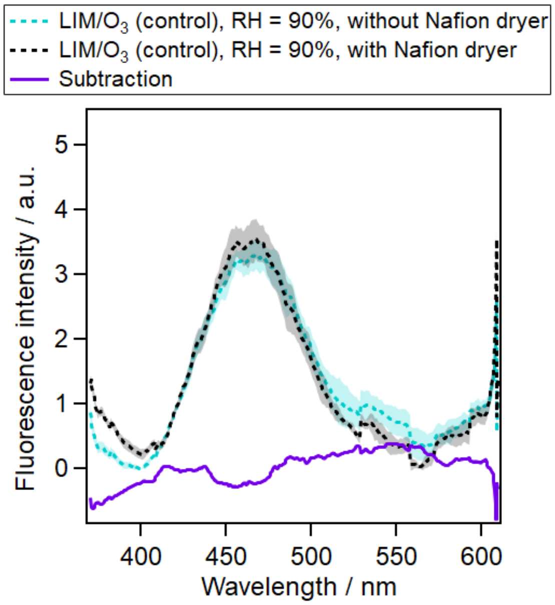

Figure S6. Fluorescence spectra of LIM/O_3-generated SOA particles without/with a Nafion dryer connected in front of the S2FS. The fluorescence spectra were averaged over 4 groups of particles and smoothed over 64 pixels. Each group consists of 5000 particles. The shades areas indicate standard deviations. No obvious change was observed for the fluorescence spectrum without a Nafion dryer and with a Nafion dryer. Purple lines (subtraction) show the fluorescence without a Nafion dryer minus that with a Nafion dryer.

Reference:

1. Zhang, M.; Klimach, T.; Ma, N.; Könemann, T.; Pöhlker, C.; Wang, Z.; Kuhn, U.; Scheck, N.; Pöschl, U.; Su, H.; Cheng, Y. Size-resolved single-particle fluorescence spectrometer for real-time analysis of bioaerosols: laboratory evaluation and atmospheric measurements. *Environ. Sci. Technol.* **2019**, *53* (22), 13257–13264.
